# Are We a Nation of Liars?

**Published:** 1888-12-01

**Authors:** 


### The Hospital

A Weekly Institutional Journal of

Science, Medicine, Nursing, and Philanthropy.

*For Hospitals, Asylums, Medical Practitioners, Students, Nurses, Families, Parishes, Congregations, and the Charitable Public.*

Vol. V. No. 114.] [December 1, 1888.

### Are We a Nation of Liars?

Are we a nation of liars? The question is suggested by a long letter which appeared in the *Times* of November 10th, under the heading, "The Increase of Mendacity," and over the signature of "Philalethes." It is equivalent to another question, namely this, "Are we a nation of scoundrels?" For if a man be such that he can properly be labelled "liar," he is capable of any kind of villany which may be practised without danger to his neck, and therefore "scoundrel," which is somewhat more comprehensive than "liar," would be a more correct and descriptive hall-mark for him. If this be so, it is evident that the inquiry we have set ourselves cannot by anyone but a fool be entered upon with a light heart. Anonymous writers to newspapers are generally regarded as somewhat shadowy personalities. But though "Philalethes" be vague and ghostlike to the reader, he is a very concrete reality to the responsible conductors of the *Times*. It need not be doubted that he is entitled to speak. The fact that he has had assigned to him more than a column of leaded type is quite sufficient to settle that question. Besides, the letter itself bears internal evidence of the experience and trained capacity of the writer. "Philalethes" starts with the assumption that "lying," or "mendacity," as he more euphoniously calls it, is on the increase. It is better to call it "lying" at once; and "Philalethes" is apparently convinced that in this country, including England, Ireland, Scotland, and Wales, this lowest, cowardliest, and meanest of vices is on the increase.

Now a man does not run up against one of his fellow-men in the street and publicly and deliberately brand him as a scoundrel without reason. At least, he does not so behave unless he is a lunatic. Neither will a sober and thoughtful person proclaim to all the world in the pages of a newspaper, whose readers are in every clime, the fact of his fellow-countrymen's degradation without what he considers a sufficient cause. "Philalethes" gives reasons, which at any rate appear convincing to himself. Politicians in times of peace fill the largest space in the public eye. The first, therefore, to be placed in the pillory by "Philalethes" are, very properly, politicians. Those gentlemen, it is said, have departed widely from the standard of veracity maintained by their predecessors. Nay, more; the reputation for truthfulness, which politicians formerly enjoyed, is only valued by their successors because it enables them the more completely to abuse the credulity of the public. "The most monstrous inventions" are not only palmed off on greedy audiences by these degenerate legislators, but even after those inventions have been over and over again exposed, they are over and over again repeated with the most unblushing effrontery.

All this sounds very shocking. The Houses of Parliament must surely swarm with infamous Catilines, who are conspiring against the morals of the State, whilst there is no indignant Cicero to declaim against the manners of the times. But is "Philalethes" justified by facts and history when he proclaims so loudly that the former days were better than these? Most emphatically not! He promulgates, honestly enough, statements and affirmations which the merest tyro in historical study can straightway overthrow. We shall not here drag forward the names of politicians and other public men who during the last two or three centuries have made themselves notorious by lying, bribery, theft, perjury, and scoundrelism of all kinds, which if done by private persons would have covered them with infamy to the latest generations. The shortest way with "Philalethes" is to ask him to give names, dates, and precise particulars of the political liars of the present generation. Then let the persons named be compared in that and every other detail of their public conduct with the statesmen of the last ten generations. If the comparison be not absolutely in favour of modern politicians, then every historian from Hume to Hallam, and from Clarendon to Green, has wilfully or ignorantly deceived the people.

From the politicians to the newspapers is but a step. There are certain of these it is said, which subsist upon "personal tittle-tattle." Lying is their stock-in-trade. They garnish falsehoods as the cook arranges parsley round the cold beef, or as the French *chef* dishes up woodcock on toast. Never was there such a generation of vipers. Oh "Philalethes!" Can it be possible that you have forgotten the pamphlets, the lampoons and the squibs, political and personal, of former generations? The stories, the society gossip, the personal tittle-tattle of to-day is as rose water compared with the dirt, the filth, the shamelessness of some periods of our history. We do not admire the so-called society papers—far from it! Some of them are so incomparably stupid that the most highly-spiced lies that were ever published could not make them the least bit interesting. But neither their stupidity nor their mendacity can at all compare with the worthlessness of similar publications a hundred years ago.

The "spirited advertiser" is the next culprit arraigned at the bar of public opinion by "Philalethes." His assertions about his wares are said to be generally "not only untrue but impossible." Adulteration it is declared, is carried to a hitherto unparalleled extent. Great capitalists, aided by the latest discoveries in chemical and mechanical science, find a chief source of profit in this dastardly method of theft. It is perhaps true that adulteration is practised by some people with more skill than formerly, though it is quite certain many of our fathers only did not adulterate because they did not know how. We are not concerned to defend adulteration. It is incapable of defence when practised under the cloak of fair dealing. But all "mixing" must not be classed together under the opprobrious name of "adulteration." If mixed wares are wholesome, and are sold as mixed, where is the knavery? That, however, is not just now the point. Our contention is that there is to-day at least as much honest business carried on as there has been in any previous age. First-rate prices still command goods of first-rate quality; and although there may be now in the world's market a cheap class of wares which were formerly unknown, yet many of the best English goods are still pre-eminent over all their rivals in almost every country in the world. No doubt advertisers puff their wares. But we are informed on good authority that no amount of puffery will persuade the public that a bad article is good, or that a dear one is cheap.

"Philalethes" claims that science has taught us to speak the truth. Is that really so? We admit gladly, proudly, that science in her efforts is truth itself. Modern science, much more than ancient science, is full of the nobility of simplicity and truth. But is it either truthful, or candid, or wise, or right to rob one king in order to crown another? Has religion—above all the Christian religion—never had anything to say about truth and simplicity? Who was it said, "Let your yea be yea, and your nay, nay; for whatsoever is more than these cometh of evil." Did science ever teach more simplicity, more truthfulness than that? We admire science; but we mean by science, all science—the science of body, the science of mind, the science of soul; material science, mental science, moral science. We admire exactness too; but a mere mechanical exactness, instead of being always truthful, may be as false as false can be. Is the mathematician true, and is the poet false? Is gravitation scientific, and is music a delusion? Science is true; but poetry, too, is true; and music and religion. The man of one idea has his uses; but he is working in the molemound of a limited specialism. He is still greater who sees all the world than he who sees one planet and calls it the world.

It is admitted that this age has many faults; that liars abound even among the great and powerful; that cheats are numerous even among the rich and prosperous. But what says Science on the topic? Does she not say: "Beware of sweeping generalisations; take care of your inductions; stick to your facts; look at both sides of the question; do not be imposed upon by mere appearances. Facts, facts, and evermore facts must be both the foundation and the superstructure of your argument." Keeping closely to observed facts we venture to affirm, as the result of a not very limited experience, that Great Britain is still at heart and in the main honest and truth-loving; animated by noble ideals, inspired by great purposes; and if her people can but join hands of true brotherhood with each other, Providence will yet give to us and our successors, as to our fathers, a place among the greatest of the nations.

